# Diseases of the Blood

**Published:** 1904-10-22

**Authors:** 


### PROGRESS IN MEDICINE AND SURGERY.

### DISEASES OF THE BLOOD.

(*Continued from page* 51.)

**Pernicious Anæmia.**—T. Law Webb[12] discusses Ehrlich's dictum that the presence of megaloblasts with a high colour-index is a conclusive test of pernicious anæmia, and concludes that it is true with very rare exceptions. A few cases of worm infection indeed may show megaloblasts and a moderately high colour-index, and cases of cancer when the bone-marrow is involved may also comply with the test, but such conditions are very rare. A high colour-index without megaloblasts is certainly not pathognomonic, and may be found in Raynaud's disease and other states, but great care is needed not to overlook these cells if they are few in number. A modification of Durham's automatic pipette is recommended for taking up an exact amount of blood in making films. C. G. Stockton[13] has investigated the frequence of gastric troubles in pernicious anæmia with reference to W. Hunter's theories. In 24 cases 18 had stomach disturbance, all had some irregularity of the bowels, 15 showed tongues denuded of epithelium. In 9 there was gastric dilatation, in 13 there was no gastric digestion, and in 8 very little, while in 5 there was gastric catarrh. The average acidity of the gastric contents was only 10, and in 21 there was no free HCl. Cabot remarks upon this that in 150 cases of pernicious anæmia, 128 had no HCl, which is certainly a remarkable figure. Some writers mention great improvement when large doses of HCl are given after meals. Reuling[14] describes the changes in the spinal cord which have been previously recognised in this disease. In two cases these changes consisted in degeneration of the posterior tract, and in another there were hæmorrhages and consequent areas of degeneration in the anterior parts of the cord. Lichtheim and others have referred to the tabetic conditions produced in some patients. Mental symptoms, even insanity, have been recorded. W. Pickett[15] finds the type is one of shallow confusion with impairment of the ideas of time and space. Hallucinatious may appear and delusions of persecution, but rarely does anything like mania occur. If Hunter's theory be correct there should be a serum capable of acting favourably in pernicious anæmia. J. Walsh and others have used streptococcic serum with varying results. Walsh's patient, however, had no visible mouth lesions, and had been particularly careful as to the cleanliness of her teeth, which were in thoroughly good order. The treatment failed. Possibly the other agent of infection was all-important here, or the streptococcus belonged to a different strain, unaffected by the particular serum used. If we could distinguish the various strains of streptococci there would, perhaps, be fewer failures from the antistreptococcal serum.

**Banti's Disease.**—Chenisse[17] holds that this disease presents three stages. (*a*) Anæmia with splenic enlargement but no other abnormality, lasting for from three to five years. The anæmia is of the secondary type. The diminution of corpuscles is variable in degree, but not so great as that of the hæmoglobin. There is generally leucopenia and a relative diminution of the finely granular eosinophils. (*b*) An intermediate stage, when gastro-intestinal troubles begin and there is urobilinuria and yellow tinting of the skin, with, also, excessive secretion of urates. (*c*) The ascitic stage, in which a rapidly developing atrophic cirrhosis of the liver leads to great distention of the abdomen by fluid. The progress of the disease is now rapid, and death generally occurs within a year of the onset of ascites. This stage, unless the splenic tumour is recognised, very much resembles alcoholic cirrhosis of the liver. The etiology is obscure. Malaria, syphilis, and alcohol are excluded as causative factors, and no results have been obtained by inoculation with the spleen pulp or blood. The histopathology indicates a toxic process acting first on the spleen, later on the blood and body generally, and finally causing a development of fibrous tissue round the portal veins of the liver and a condition of atrophic cirrhosis. The most important point in connection with this disease is that splenectomy may cure, even after cirrhosis of the liver has began to develop. Dock and Warthin[18] report two cases of Banti's disease. In one a man, aged 41, unfortunately died three days after having had his spleen removed. The autopsy fully justified the diagnosis on which the advice was founded. There was early interstitial hepatitis and stenosis of the portal vein at its commencement just after the union of the splenic with the mesenteric vein. Fibrous bands and valve-like constrictions almost occluded its lumen, so that it would barely admit the tip of a large probe. The spleen showed evidence of chronic passive congestion, hyperplasia of the reticulum, and atrophy of the lymphoid elements. There was increase and activity of the lymphoid tissues in the abdomen, indicating a compensatory hypertrophy, these structures probably taking on the function of the spleen. A curious point in the family history is that the patient's wife had died of leukæmia a year before his illness definitely began and he had himself shown great interest in splenomegaly, introducing other cases of the kind to the hospital. The second case—a woman—was not sufficiently clear to warrant the diagnosis of Banti's disease during life. There was pelvic trouble which masked the other, and, in accordance with the patient's strong wishes, an operation was performed. She recovered well, but the ascites returned rapidly and she died of gastric hæmorrhage rather more than a month later. The pathological changes were similar to those in the first case, including the same high degree of stenosis of the commencement of the portal vein. Whether this was primary or arose along with the fibrosis of the spleen from a general toxic action falling especially on this area, or whether the toxin originated in these cases in the spleen, the authors cannot say, but they agree with other writers in regarding the splenic disease as being of longer standing than the hepatic cirrhosis. They also draw attention to the compensatory hæmolytic function exercised by the hæmo-lymph nodes. Warthin's study of those structures in these and other cases are of considerable interest.

**Leukæmia.**—Lockart Gillespie[19] gives a valuable summary of recent cases which show the possible variations from the two main types. In Billing's and Capp's paper we have an acute case of the spleno-medullary type, the existence of which has been denied, perhaps owing to the difficulty of distinguishing large lymphocytes from myelocytes. Six other undoubted cases of the same kind are on record. Kelly comes to the conclusion that both lymphatic and spleno-medullary leukæmia are derived from disease of the bone marrow, though the cells found differ so widely, probably from a difference in the poison which causes the proliferation. To confirm this view of the unity of origin of the two types Reed analyses a case of the acute lymphatic form where there was no glandular enlargement, showing that the lymphocytes arose from the lymphoid cells of the marrow. Hamman has collected accounts of 124 cases of acute leukæmia, of which the great majority were of the large lymphoid type, and he too thinks that these large cells are derived from the marrow. L. B. Goldhorn[20] holds that myelogenous leukæmia is a disease of those centres of the marrow which supply granular cells to the blood, while the lymphatic form is a disease of those centres which supply lymphocytes. Both can form metastases like cancer does. A mixed leukæmia is probably as rare as carcinoma with sarcomatous elements in it. The apparent cases are due to the presence of large mononuclear hyaline cells which are not really lymphocytes, but a type of true marrow cells. A case recently recorded[21] helps to fill the gap in our knowledge of the initial stages of this disease. The diagnosis, if the case had come under observation only in its more pronounced stage, would probably have been "Acute Leukæmia," but the writer watched through 17 months a gradually increasing anæmia with low colour-index, with little or no leucocytosis and at first no enlargement of the liver, spleen or glands, but a persistently high and increasing percentage of myelocytes, lymphoid cells and nucleated reds. The spleen became palpable some six months before death, but was not much enlarged until a week before the patient died, when a gangrenous stomatitis set in, with petechial eruptions. Up to this point the patient had been fairly well, and had gone about his business. During the last week the spleen reached to the umbilicus, the liver was enlarged, and so also were the glands in the neck. The gums bled, there were much nausea, some vomiting, continuous high fever, and the end was in coma. The leucocytes at the beginning of the week were 116,000, myelocytes 46 per cent., and large hyalines 30 per cent. Throughout the whole of the long series of blood-examinations only two coarsely granular eosinophils were found, one a myelocyte and one a leucocyte. Evidence is accumulating that the curious and rare condition of *chloroma* has some intimate relation with the Leukæmias. In fact, except that the deposits in the organs are of a bright green colour, the clinical and pathological picture is almost precisely that of lymphatic leukæmia, including that symptom, so very common in the early stages of leukæmia, an obstinate sore throat. Certain curious features distinguish chloroma; for instance, exophthalmos, due to deposits in the orbital bones, and headache and deafness, probably from a similar cause in the bones of the skull. It was formerly regarded as a sarcomatous condition, but we must give up that classification so long as we deny a place among the sarcomas to leukæmia. Recent cases are reported by Byrom Bramwell, Dock,[22] and others. A case of *multiple myeloma*, with Bence-Jones proteid in the urine[23] occurred in the German hospital at Dalston. The symptoms complained of were of a rheumatoid character, and when the discovery of the state of the urine revealed the rare condition, they had lasted about six months. Death occurred six months later. The patient had had syphilis and gonorrhœa. The peculiar proteid which is found in these cases is characterised by being precipitated when heated to a low temperature, by being partially redissolved on boiling, and completely redissolved on the addition of acid. It reappears on cooling. These phenomena occur in acid urine. The patient in this case regularly passed about seven parts per 1,000 up to the time of his death. At the autopsy there was found a diffuse growth in the shafts of the long bones of which Professor R. Muir said "that the tissue was formed from a special and characteristic type of cell which was probably derived from the neutrophil myelocyte or its predecessor; that the cell produced in its protoplasm, in a granular form, a substance which was closely allied to, though not identical with, the substance of the neutrophil granules of the myelocytes." He suggested the possibility of the relationship between the formation of the granules in the growth and the excretion of Bence-Jones proteid. The author considers that it has no relation to food, as considerable changes of diet produced no alteration in its excretion. He regards it as a product of the action of a digestive enzyme on the circulating proteid of the blood.

12 Birmingham Med. Rev., July. 13 Med. Rec., June 11. 14 Amer. Jour. Med. Sc., March. 15 Ibid., June. 16 Med. Rec., Feb. 26. 17 La Semaine Med., vol. xxxvi. p. 301. 18 Dock and Warthin, Amer. Jour. Med. Sc, Jan. 19 Edin. Med. Jour., June. 20 Med. Rec., April 23. 21 Amer. Jour. Med. Sc., June. 22 Amer. Jour. Med. Sc., vol. cvi., p. 152. 23 Jour. of Path. and Bac., Dec.

(*To be concluded.*)

---

